# Prognostic value of early point-of-care blood gas analysis in critically ill non-trauma patients

**DOI:** 10.1186/s12873-026-01822-2

**Published:** 2026-10-21

**Authors:** Constantin Maier-Stocker, Rebekka Läng, Jillena Zinsser-Krys, Frank Hanses, Markus Zimmermann, Julian Hupf

**Affiliations:** 1 Department of Emergency Medicine, University Hospital Regensburg, Regensburg, Germany; 2 Department for Infection Control and Infectious Diseases, University Hospital Regensburg, Regensburg, Germany

**Keywords:** Blood gas analysis, Critically Ill, Prognosis, Emergency department, Point-of-care

## Abstract

**Background:**

Critically ill non-trauma patients treated in the emergency department have a high risk of in-hospital mortality. While point-of-care diagnostics such as blood gas analysis are established tools for urgent management, data on their prognostic value in this specific patient cohort remain limited. This study aimed to evaluate whether initial blood gas parameters are independently associated with in-hospital mortality in critically ill non-trauma patients.

**Methods:**

This retrospective cohort study included adult critically ill non-trauma patients treated in the emergency department of a German university hospital. Initial blood gas parameters and clinical data were analyzed using univariate and multivariable logistic regression to assess their association with in-hospital mortality.

**Results:**

Of 879 screened patients, 542 met the inclusion criteria. In-hospital mortality was 20% (*n* = 110). In addition to older age and out-of-hospital cardiac arrest, anemia (hemoglobin < 12 g/dL; OR of 2.07, 95% CI 1.20–3.58; *p* = 0.009), acidosis (pH < 7.35; OR 2.26, 95% CI 1.09–4.95; *p* = 0.033) and hypobicarbonatemia (< 22 mmol/L; OR 2.79, 95% CI 1.42–5.69; *p* = 0.004) were independently associated with in-hospital mortality. The association for hyperlactatemia was borderline (> 2.2 mmol/L; OR 1.85, 95% CI 0.93–3.77; *p* = 0.08). Multivariate analysis did not reveal significant associations between other electrolyte imbalances and in-hospital mortality in this cohort.

**Conclusion:**

In this retrospective cohort, the presence of anemia, acidosis or hypobicarbonatemia on admission to the resuscitation room were independently associated with an elevated risk of in-hospital mortality in critically ill non-trauma patients. These readily available point-of-care parameters may support early risk stratification. Prospective studies are needed to validate these findings.

**Supplementary Information:**

The online version contains supplementary material available at https://doi.org/10.1186/s12873-026-01822-2 .

## Introduction

The management of critically ill non-trauma patients in the emergency department presents a significant challenge, with high in-hospital mortality rates among this patient population. Consequently, it is well established that critically ill patients should be treated in a specialized treatment area within the emergency department, commonly referred to as resuscitation room [1].

Early identification of high-risk patients is crucial for guiding subsequent management and improving outcomes. Management protocols assist in guiding diagnostic measurements and treatment [2]. Point-of-care diagnostics such as blood gas analysis offer rapid availability, reliability, and cost-effectiveness. Therefore, they are frequently used in time-sensitive contexts, such as the emergency management of critically ill patients admitted to the resuscitation room. Existing evidence from critically ill patients in the emergency department indicates that hyperlactatemia at admission is associated with increased mortality and can assist in identifying high-risk patients [3]. Nevertheless, comprehensive data on the prognostic value of blood gas parameters for critically ill non-trauma patients treated in the resuscitation room remain limited. The primary aim of this study was therefore to evaluate whether blood gas parameters are independently associated with in-hospital mortality in these patients.

## Methods

### Study population

This retrospective cohort study was conducted at the Emergency Department of the University Hospital Regensburg, Germany. It included all adult (≥ 18 years), critically ill non-trauma patients (triaged with an Emergency Severity Index [ESI] of I or II) treated in one of the resuscitation rooms of the Emergency Department of the University Hospital Regensburg, Germany, between January 1, 2016 and August 31, 2017. ESI is a five-level triage tool, with ESI I and II indicating the most critically ill patients requiring immediate or very urgent treatment. The Emergency Department of the University Hospital Regensburg is a tertiary care center with a dedicated resuscitation room for critically ill patients. Approximately 52,600 cases were treated in the Emergency Department during the study period. Allocation to the resuscitation room was determined by the emergency medical services based on the presence of at least one immediately life-threatening condition, including airway compromise, respiratory failure, haemodynamic instability, or a critical neurological emergency.

Patients were excluded if they deceased in the resuscitation room, if treatment was limited to best supportive care, if they were transferred to another hospital before the in-hospital outcome could be determined, or if documentation was insufficient for retrospective analysis (defined as the inability to reliably retrieve key variables from the electronic medical records, e.g. initial blood gas parameters or discharge outcome). Because the primary endpoint was in-hospital mortality, the exclusion of resuscitation-room deaths and transferred patients may induce a survivorship or selection bias. The primary analysis should therefore be interpreted as estimating the prognostic association of the initial blood gas among patients who survive the initial resuscitation-room phase and for whom the in-hospital outcome is ascertainable, rather than among the entire presenting population. The impact of excluding early deaths was assessed in a pre-specified sensitivity analysis that included resuscitation-room deaths with an available initial blood gas measurement.

This study was conducted and reported in accordance with the STROBE guidelines [4].

### Data

In addition to baseline patient characteristics, including age and sex, the following clinical data were collected from the electronic medical records: the primary diagnosis established after resuscitation room treatment, the final hospital discharge diagnosis, out-of-hospital cardiac arrest (OHCA) status, intensive care unit (ICU) admission, and duration of the ICU stay. Presentation diagnoses reflect the primary diagnosis assigned by the treating emergency physician at the end of the resuscitation phase, and were classified by organ system or etiology.

The parameters of the first blood gas analysis obtained in the resuscitation room and routine laboratory values were also recorded. Both venous and arterial blood gas samples were analyzed with equal weighting, as previous literature has demonstrated acceptable clinical concordance between these sampling methods for the parameters assessed [5–10]. Partial pressure of oxygen (pO_2_) was excluded because venous and arterial measurements are not interchangeable. Partial pressure of carbon dioxide (pCO_2_) was not a primary metabolic marker because its interpretation is closely tied to ventilatory status, which was not consistently documented.

To enhance clinical applicability continuous blood gas variables were categorized using established clinical reference ranges (pH 7.35–7.45; standard bicarbonate 22–28 mmol/L; base excess − 2 to + 2 mmol/L; sodium 135–145 mmol/L; potassium 3.5–5.5 mmol/L; calcium 1.15–1.35 mmol/L; chloride 96–110 mmol/L; hemoglobin 12–18 g/dL). For glucose we used the mortality-associated breakpoints reported by Bernhard et al. [11] in critically ill emergency patients (normal 100–272 mg/dL; low < 100; high > 272 mg/dL) rather than a range derived from non-critically-ill populations. Lactate was categorized as elevated above the point-of-care analyzer’s reference limit of 2.2 mmol/L (20 mg/dL). Values below the lower reference limit were classified as “low” and those above the upper limit as “high”. In-hospital mortality was evaluated as the primary outcome through a retrospective analysis of the patients’ discharge records.

### Statistical analysis

Analyses were performed in R version 4.5.3 (R Foundation for Statistical Computing, Vienna, Austria), using the rms package for restricted cubic splines and internal bootstrap validation, pROC for ROC analysis, and car for collinearity diagnostics. Categorical variables are presented as absolute numbers and frequencies (%), continuous variables are reported as medians with interquartile ranges (IQR). The threshold for statistical significance was two-sided *p* < 0.05. Univariate associations with in-hospital mortality were assessed using Pearson’s Chi-squared test for categorical variables and the Mann-Whitney U test for continuous variables and are reported descriptively. As these univariate analyses were considered exploratory and served as descriptive screening, p-values were not adjusted for multiple testing. Age, sex, and OHCA status were included as covariates on clinical grounds, being established prognostic factors. The initial blood gas parameters were first screened individually against in-hospital mortality. Base excess was not carried forward because of collinearity with bicarbonate, and sodium, potassium, and chloride were not carried forward as they showed no univariate association with mortality. The multivariable logistic regression model therefore comprised age, sex, OHCA, and the remaining blood gas parameters pH, bicarbonate, lactate, glucose, calcium, and hemoglobin. The model was fitted by complete-case analysis, no imputation was performed. Odds ratios (OR) are reported with 95% confidence intervals (CI).

Multicollinearity was assessed with variance inflation factors (generalized VIF for multi-level factors) and pairwise correlations among the continuous acid-base variables. Discrimination was quantified by the c-statistic (area under the ROC curve) with bootstrap optimism correction (200 replications). Calibration was evaluated with the Hosmer-Lemeshow test and a calibration plot, and the Nagelkerke R^2^ is reported.

As dichotomization loses information and assumes an abrupt change in risk at the cut-off, the categorical model was supplemented by analyses on the continuous data. Each parameter modelled as a continuous predictor, and restricted cubic splines (four knots) to test for non-linearity. A further sensitivity analysis re-included patients who deceased in the resuscitation room but had an available initial blood gas. In addition, mortality was tabulated across graded pH and glucose strata with a test for trend to assess a dose-response relationship. The chosen cut-offs were applied for clinical applicability and were not intended as validated prognostic thresholds.

## Results

**Fig. 1:**
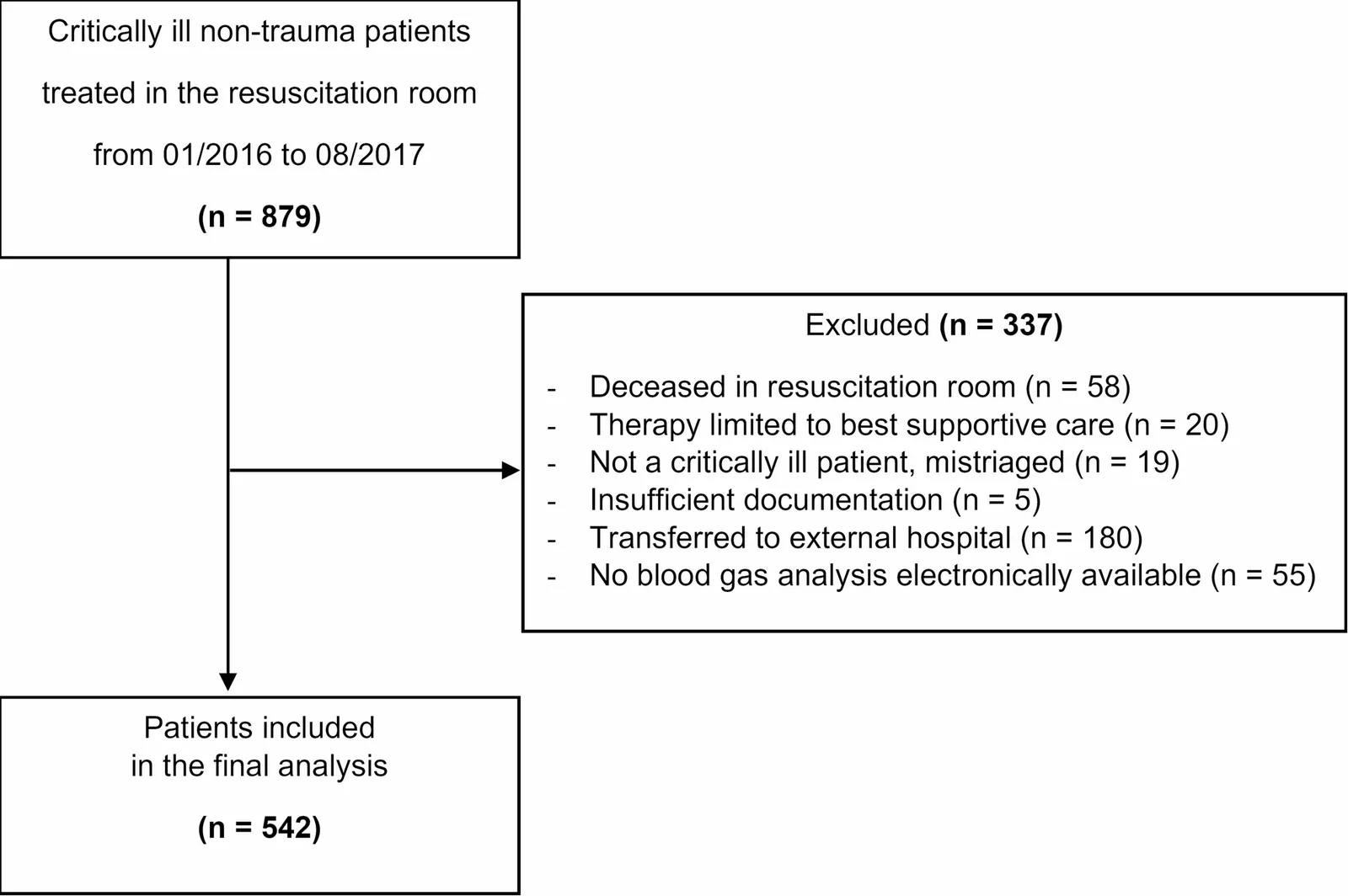
Flow diagram of patient inclusion and exclusion

Of 879 patients treated in a resuscitation room during the study period, 542 (61.7%) met the inclusion criteria. The inclusion and exclusion process is illustrated in Fig. 1.

### Patients

The characteristics of the patients included in the study are presented in Table 1. Most patients presented with cardiac disease (48%), respiratory disease (15%), or intoxication (12%) as the underlying diagnosis. The in-hospital mortality rate was 20% (*n* = 110).

**Table 1:** Baseline characteristics

Characteristic	*n* = 542	%; IQR
**Age (years)**,** median**	64	51–74
**Age groups**		
< 60 years	229	42%
≥ 60 years	313	58%
**Sex**		
Male	372	69%
Female	170	31%
**OHCA**	166	31%
**ICU admission**	426	79%
**Deceased**	110	20%

Data are presented as absolute numbers with percentages for categorical variables and as median with interquartile range [IQR] for continuous variables. OHCA: out-of-hospital cardiac arrest; ICU: intensive care unit

### Blood gas parameters

Table 2 outlines the initial blood gas parameter distributions of the study population upon admission to the resuscitation room. The most prevalent abnormalities were unbalanced base excess and abnormal pH, followed by elevated lactate and abnormal bicarbonate.

**Table 2:** Distribution of initial blood gas parameters upon admission

Parameter	*n*	%	Parameter	*n*	%
**pH**	542		**Hemoglobin**	542	
7.35–7.45	179	33.0	12–18 g/dL	388	71.6
< 7.35	332	61.3	< 12 g/dL	149	27.5
> 7.45	31	5.7	> 18 g/dL	5	0.9
**Base excess**	541		**Sodium**	542	
-2-2 mmol/L	167	30.9	135–145 mmol/L	427	78.8
< -2 mmol/L	290	53.6	< 135 mmol/L	83	15.3
> 2 mmol/L	84	15.5	> 145 mmol/L	32	5.9
**Bicarbonate**	537		**Potassium**	542	
22–28 mmol/L	248	46.2	3.5–5.5 mmol/L	427	78.8
< 22 mmol/L	266	49.5	< 3.5 mmol/L	85	15.7
> 28 mmol/L	23	4.3	> 5.5 mmol/L	30	5.5
**Lactate**	541		**Calcium**	541	
≤ 2.2 mmol/L	244	45.1	1.15–1.35 mmol/L	334	61.7
> 2.2 mmol/L	297	54.9	< 1.15 mmol/L	195	36.0
			> 1.35 mmol/L	12	2.2
**Glucose**	542		**Chloride**	541	
100–272 mg/dL	389	71.8	96–110 mmol/L	447	82.6
< 100 mg/dL	73	13.5	< 96 mmol/L	36	6.7
> 272 mg/dL	80	14.8	> 110 mmol/L	58	10.7

Data are presented as absolute numbers with percentages

### Association with in-hospital mortality

In the univariate analysis, pH < 7.35, base excess < -2 mmol/L, bicarbonate < 22 mmol/L, lactate > 2.2 mmol/L, glucose > 272 mg/dL, and hemoglobin < 12 g/dL were each associated with increased in-hospital mortality (all *p* < 0.001). A low ionized calcium (< 1.15 mmol/L) showed a weaker but significant association (*p* = 0.007). Full univariate results, including direction-specific tests for each abnormal category, are provided in Supplementary Table S1. Among the clinical parameters, higher age (*p* = 0.025) and out-of-hospital cardiac arrest (*p* < 0.001) were associated with higher mortality, whereas sex was not (*p* = 0.066).

**Fig. 2:**
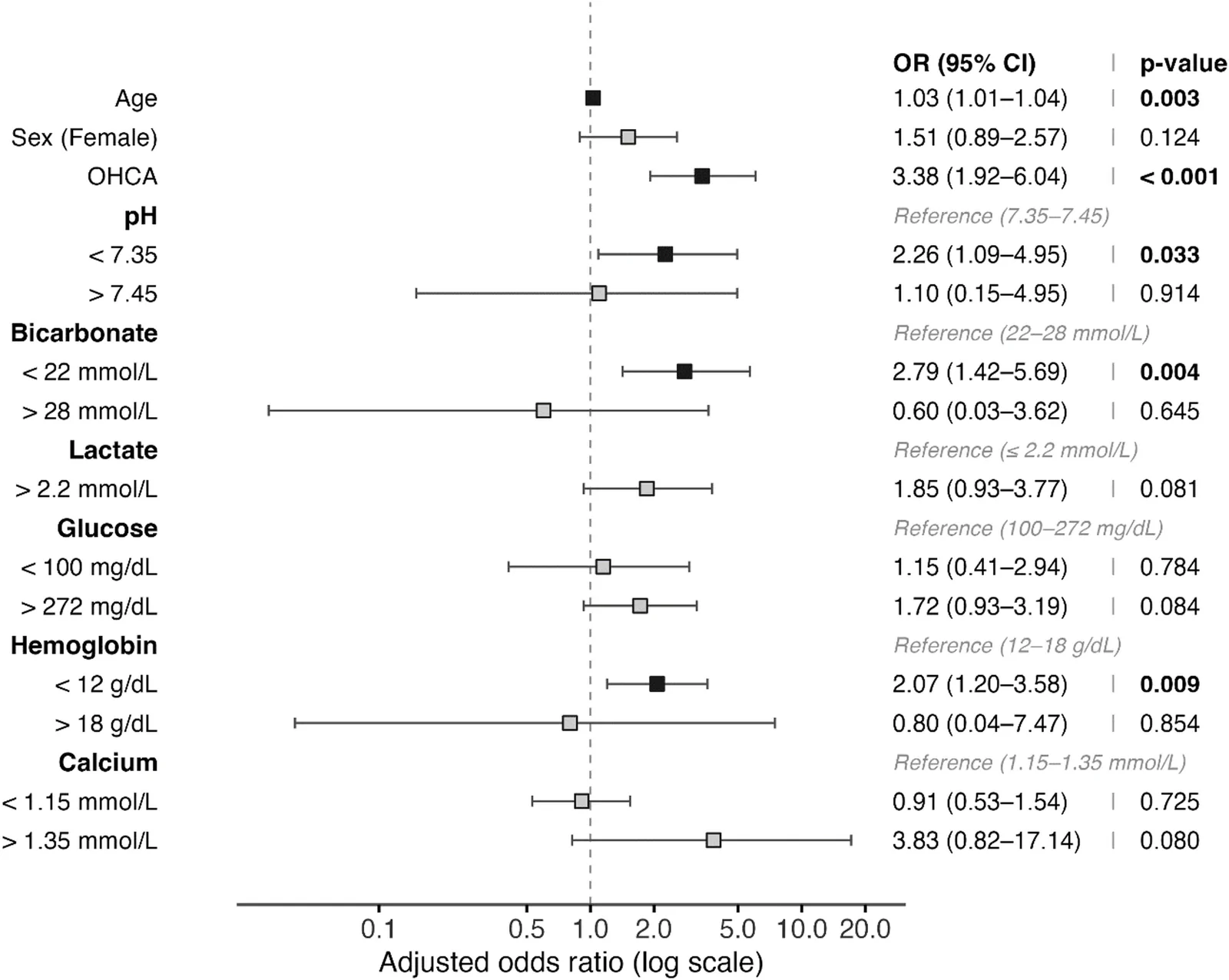
Forest plot of adjusted odds ratios for in-hospital mortality from a multivariable logistic regression model (*n* = 535) including age, sex, out-of-hospital cardiac arrest (OHCA), and the blood-gas parameters pH, bicarbonate, lactate, glucose, calcium, and hemoglobin. Blood-gas parameters were entered as categorical variables, with the normal range as reference. Filled squares indicate a 95% confidence interval excluding 1. OR: odds ratio; CI: confidence interval; OHCA: out-of-hospital cardiac arrest

After multivariable adjustment (Table 3; Fig. 2), higher age and OHCA remained associated with mortality. Among the blood gas parameters, anemia, acidosis and low bicarbonate were independently associated with increased in-hospital mortality. Elevated lactate (OR 1.85, *p* = 0.08) and hyperglycemia (OR 1.72, *p* = 0.08) showed borderline associations. Another nominal borderline association for hypercalcemia (OR 3.83, *p* = 0.08) was based on only 12 patients and was statistically unstable (95% CI 0.82–17.14). Therefore, it should be regarded as hypothesis-generating. The remaining parameters showed no association.

**Table 3:** Multivariable logistic regression: variables independently associated with in-hospital mortality (complete cases, *n* = 535)

Variable		Odds Ratio	95% CI	*p*-value
Age		1.03	1.01–1.04	0.003
Sex	Male	Reference		
	Female	1.51	0.89–2.57	0.124
OHCA	No	Reference		
	Yes	3.38	1.92–6.04	< 0.001
pH	7.35–7.45	Reference		
	< 7.35	2.26	1.09–4.95	0.033
	> 7.45	1.10	0.15–4.95	0.914
Bicarbonate	22–28 mmol/L	Reference		
	< 22 mmol/L	2.79	1.42–5.69	0.004
	> 28 mmol/L	0.60	0.03–3.62	0.645
Lactate	≤ 2.2 mmol/L	Reference		
	> 2.2 mmol/L	1.85	0.93–3.77	0.081
Glucose	100–272 mg/dL	Reference		
	< 100 mg/dL	1.15	0.41–2.94	0.784
	> 272 mg/dL	1.72	0.93–3.19	0.084
Hemoglobin	12–18 g/dL	Reference		
	< 12 g/dL	2.07	1.20–3.58	0.009
	> 18 g/dL	0.80	0.04–7.47	0.854
Calcium	1.15–1.35 mmol/L	Reference		
	< 1.15 mmol/L	0.91	0.53–1.54	0.725
	> 1.35 mmol/L	3.83	0.82–17.14	0.080

Seven of the 542 patients were excluded because of missing covariate values, leaving *n* = 535 complete cases for the multivariable logistic regression model. OR: odds ratio; CI: confidence interval; OHCA: out-of-hospital cardiac arrest

### Model performance and diagnostics

**Fig. 3:**
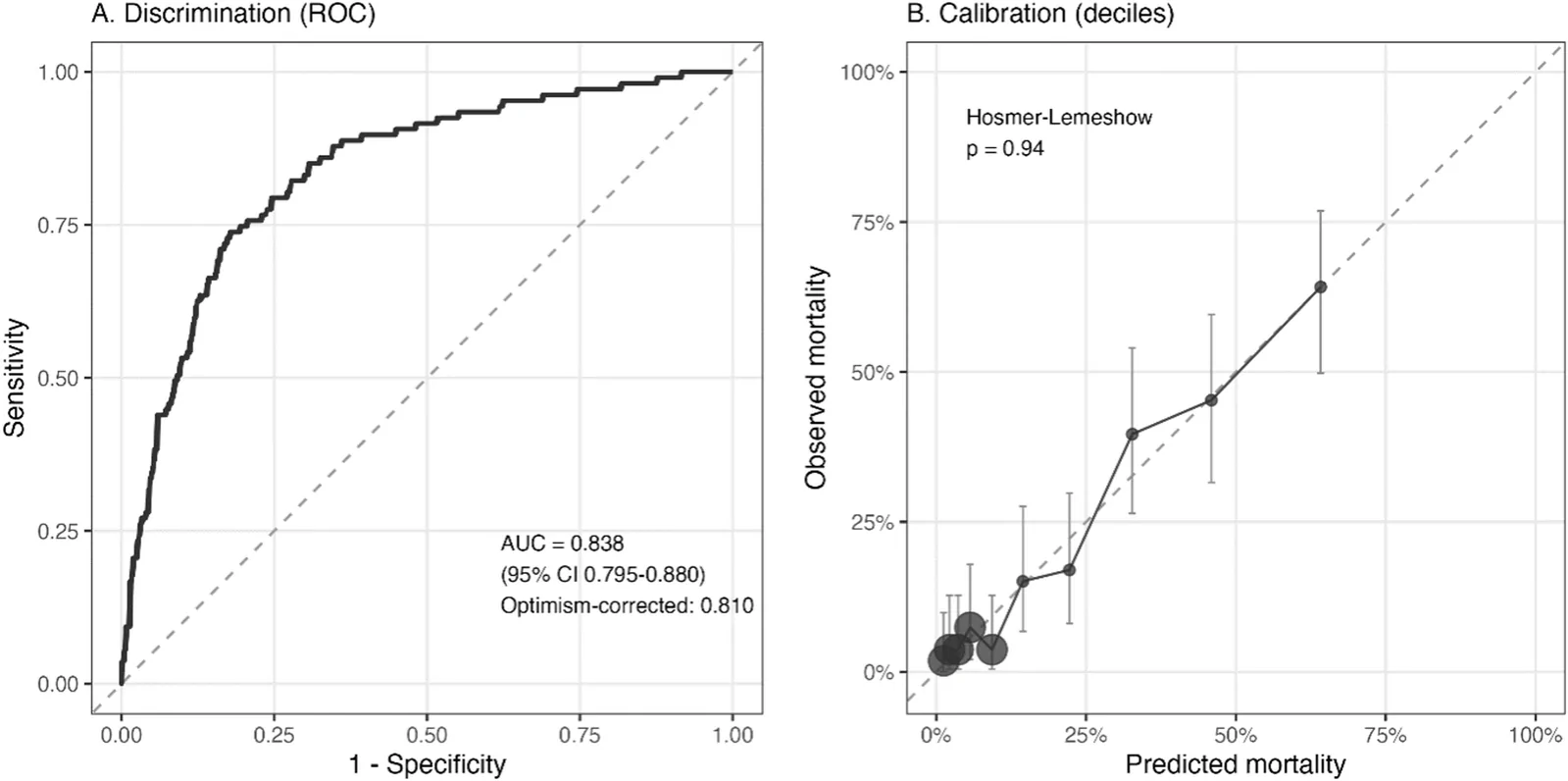
Model discrimination (ROC curve, panel A) and calibration (decile plot, panel B). In panel A, the dashed diagonal indicates chance-level discrimination. In panel B, mean predicted mortality is plotted against observed mortality across deciles of predicted risk, with the dashed diagonal indicating perfect calibration; point size is proportional to the number of patients per decile and error bars represent 95% confidence intervals for the observed mortality within each decile. AUC: area under the curve; CI: confidence interval; ROC: receiver operating characteristic

The multivariable model showed good discrimination (c-statistic 0.838, 95% CI 0.795–0.880; bootstrap optimism-corrected 0.810) and adequate calibration (Hosmer-Lemeshow *p* = 0.94; Nagelkerke R^2^ = 0.36; Supplementary Table S2). Multicollinearity was not a concern. All variance inflation factors were ≤ 1.4 (Supplementary Table S3). Among the continuous acid-base variables, the only strong correlation was between bicarbonate and base excess (*r* = 0.98). Therefore, base excess was not included in the model. The remaining correlations were moderate (pH-bicarbonate *r* = 0.75, pH-lactate *r* = -0.62 and bicarbonate-lactate *r* = -0.67; see Supplementary Table S4 and Fig. 3).

### Continuous and non-linear analysis

**Fig. 4:**
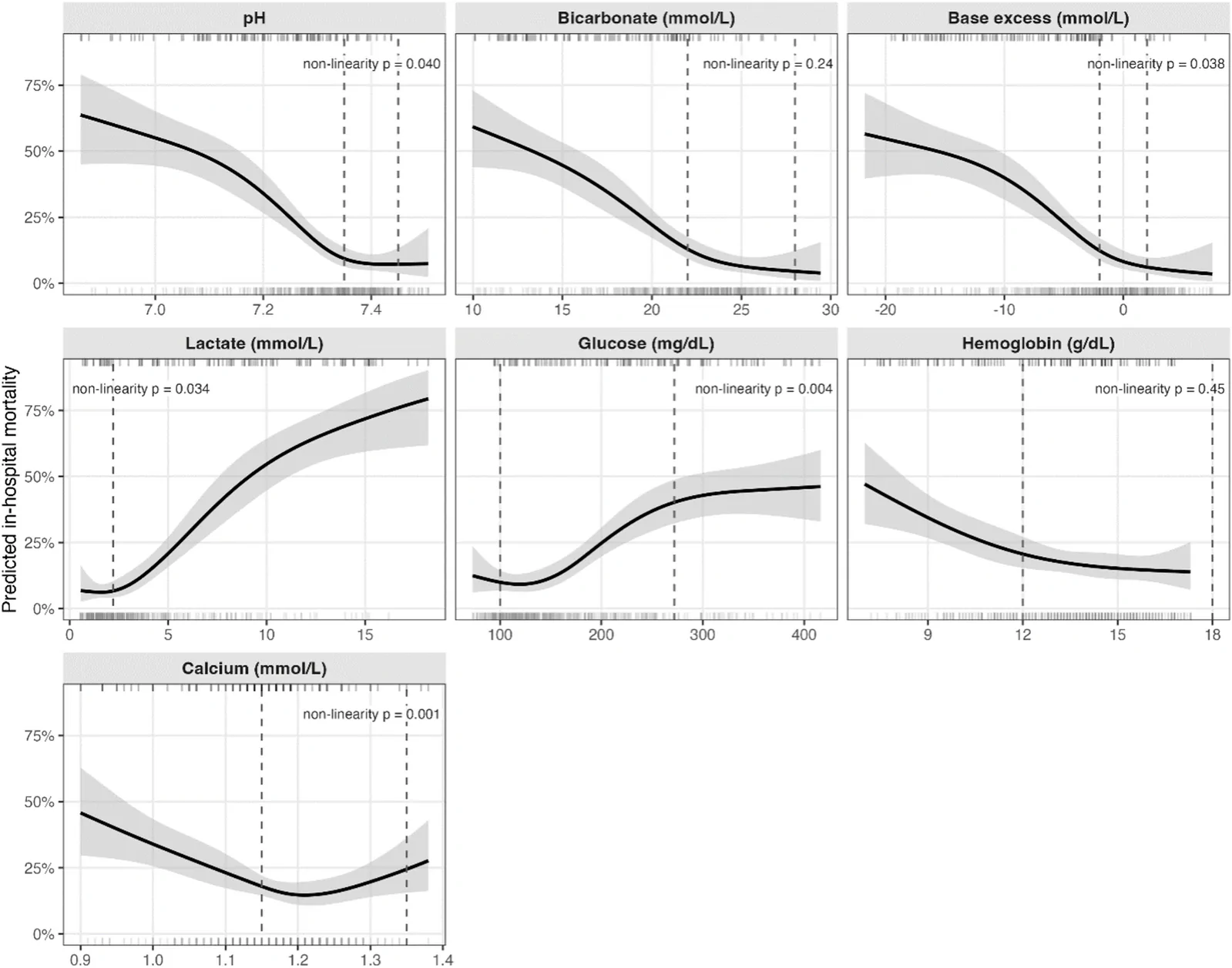
Predicted in-hospital mortality across continuous blood gas parameters. Restricted cubic splines with four knots. Axes are truncated to the bulk of the observed distribution. The solid line shows the predicted probability of in-hospital mortality and the shaded band its 95% confidence interval. Dashed vertical lines mark the reference cut-offs used for dichotomization. Rug marks indicate the distribution of observed values (deaths at the top, survivors at the bottom of each panel)

Analyzed as continuous predictors, all principal parameters were strongly associated with mortality: pH (OR 1.89 per 0.1-unit decrease, 95% CI 1.62–2.22), bicarbonate (OR 1.23 per 1 mmol/L decrease, 1.18–1.30), lactate (OR 1.33 per 1 mmol/L increase, 1.26–1.41), hemoglobin (OR 1.19 per 1 g/dL decrease, 1.10–1.29) and glucose (OR 1.46 per 50 mg/dL increase, 1.30–1.64); all *p* < 0.001. Restricted cubic splines demonstrated significant non-linearity for pH (*p* = 0.040), lactate (*p* = 0.034), glucose (*p* = 0.004; U-shaped), calcium (*p* = 0.001) and base excess (*p* = 0.038), whereas bicarbonate (*p* = 0.24) and hemoglobin (*p* = 0.45) were consistent with linear associations (Fig. 4). Lactate illustrates how dichotomization can obscure a prognostic signal. Dichotomized at 2.2 mmol/L, lactate reached only borderline significance in the multivariable model (OR 1.85, *p* = 0.081). In the unadjusted continuous analysis each 1 mmol/L increase was associated with higher odds of death (OR 1.33, *p* < 0.001), and the spline curve showed risk rising across the elevated range (Fig. 4). With the cut-off close to the cohort median (2.6 mmol/L), the binary variable places patients with mildly and markedly elevated lactate in the same category. The borderline result therefore most likely reflects the loss of information through categorization rather than the absence of a prognostic effect.

Sodium, potassium and chloride were also examined as continuous variables (Supplementary Table S5). Sodium and potassium showed no significant association with mortality, and chloride reached significance only with a negligible effect size (OR 0.98 per mmol/L). These parameters are therefore not shown in Fig. 4.

### Graded thresholds

**Fig. 5:**
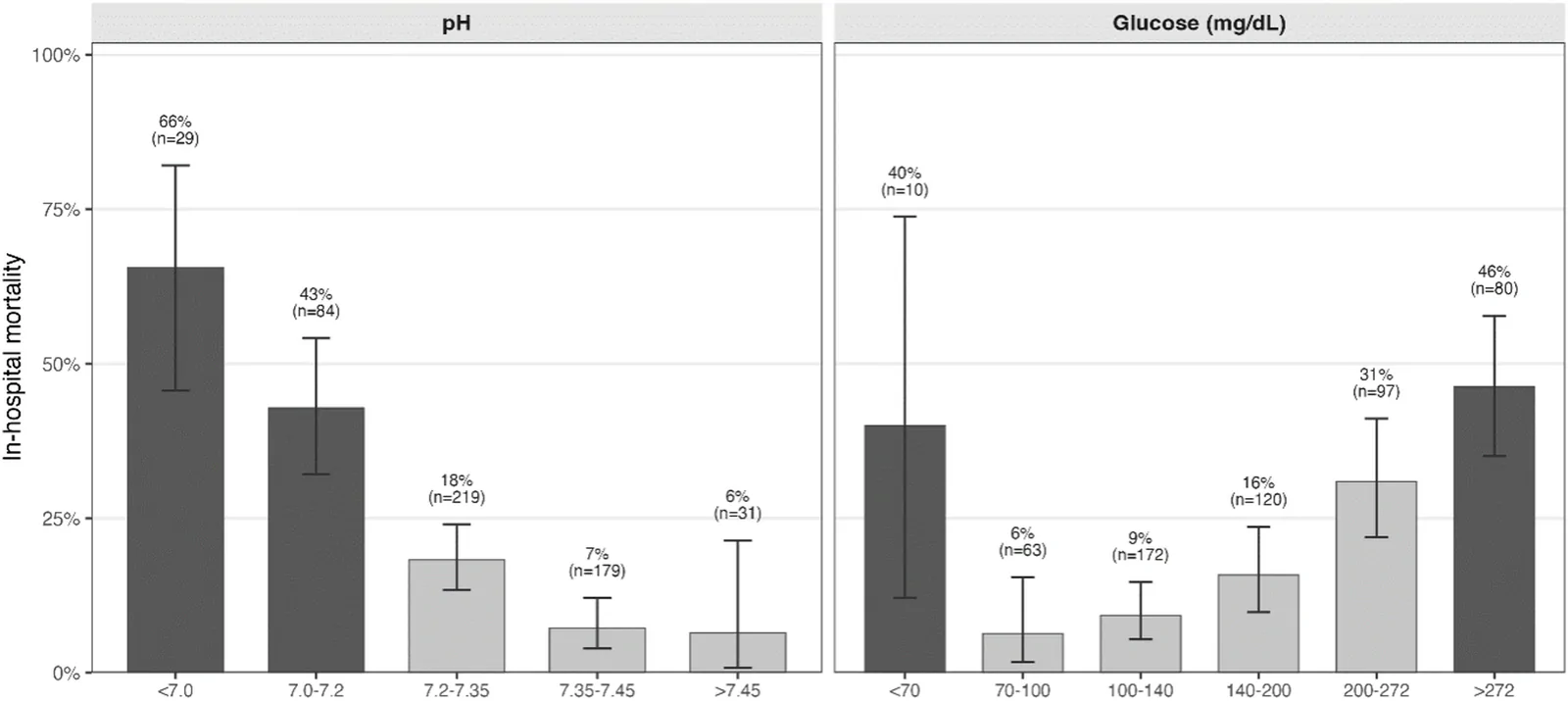
In-hospital mortality across graded pH strata (trend *p* < 0.001) and glucose strata (U-shaped, quadratic *p* = 0.006). Error bars indicate 95% exact binomial CI. Dark bars indicate strata with ≥ 40% mortality. CI: confidence interval

To complement the continuous analyses in Fig. 4 with a clinically interpretable view, we examined in-hospital mortality across graded pH and glucose strata. Mortality increased monotonically across pH strata: > 7.45, 6.5% (2/31); 7.35–7.45, 7.3% (13/179); 7.2–7.35, 18.3% (40/219); 7.0-7.2, 42.9% (36/84); and < 7.0, 65.5% (19/29) (test for trend *p* < 0.001). The steep increase below pH 7.2, and again below 7.0, indicates that severe acidosis carries a substantially higher risk than the mild, often reversible acidosis seen with some acute presentations. Glucose showed a U-shaped relationship: mortality was 40% below 70 mg/dL, fell to 6.3% at 70–100 mg/dL, and rose progressively to 46.2% at > 272 mg/dL (global *p* < 0.001; U-shaped, quadratic *p* = 0.006) (Fig. 5; Supplementary Table S6).

### Sensitivity analyses

Of the 58 patients who deceased in the resuscitation room, 13 had a complete initial blood gas panel, a consistently recorded outcome and otherwise met inclusion criteria. Re-including them (expanded cohort *n* = 555; 123 deaths, 22.2%) did not change the conclusions: OHCA (OR 4.05, *p* < 0.001), low bicarbonate (OR 2.47, *p* = 0.007), low hemoglobin (OR 1.97, *p* = 0.011) and age (OR 1.03, *p* < 0.001) remained independently associated with mortality, while the association for low pH was attenuated to borderline significance (OR 1.87, *p* = 0.081); the borderline associations for elevated lactate and hyperglycemia were essentially unchanged (Supplementary Table S7).

To confirm that the categorical findings were not an artifact of the chosen cut-offs, each parameter was additionally examined on its continuous scale and with restricted cubic splines. In the primary cohort these cut-off-independent analyses reproduced every principal association and demonstrated a significant U-shaped glucose-mortality relationship (see Continuous and non-linear analysis; Supplementary Table S5). Repeating the identical analysis in the expanded cohort yielded essentially the same estimates, with the U-shaped glucose-mortality relationship persisting (Supplementary Table S8). The dichotomized lactate association was borderline, whereas the continuous lactate association was strong, indicating that the attenuation on dichotomization reflects the loss of information inherent in categorization rather than the absence of a lactate-mortality relationship.

## Discussion

This retrospective, single-center cohort study explored the association between initial abnormal blood gas parameters and in-hospital mortality in critically ill non-trauma patients. Anemia (hemoglobin < 12 g/dL), acidosis (pH < 7.35) and low bicarbonate (< 22 mmol/L) on admission were independently associated with higher in-hospital mortality, the association for elevated lactate was borderline.

Due to the use of point-of-care diagnostics, blood gas parameters can be obtained rapidly and reliably, making them potentially valuable tools for early risk stratification.

The findings of this study are concordant with the results of a study by Georgiev et al. [12], who analyzed 1,536 non-trauma critically ill patients and identified pH as the strongest single point-of-care marker of resuscitation-room mortality (AUC 0.81), with a substantial mortality increase below pH 7.2 and a further increase below pH 7.0. The graded-threshold results of the present study reproduce this pattern closely, and the multivariable discrimination (optimism-corrected c-statistic 0.81) matches theirs. Georgiev et al. studied an independent cohort at a different center and reported the univariable discriminative performance of each marker in isolation. By their own account that analysis was exploratory and deliberately omitted a multivariable model and any examination of non-linear relationships, both of which they named as limitations and directions for future work. This study is complementary and distinct to the study of Georgiev et al. in several respects. It evaluates a broader panel of routinely available parameters within a mutually adjusted multivariable model for in-hospital mortality, quantifies the independent association of hemoglobin and the metabolic markers alongside pH, and provides a continuous/spline characterization of each parameter. It thereby addresses those open questions directly. pH, bicarbonate and hemoglobin each retained an independent association after mutual adjustment, formal collinearity diagnostics (all VIF ≤ 1.4) confirm that the instability anticipated by Georgiev et al. does not arise, and the spline analysis recovers a U-shaped glucose-mortality relationship that univariable discrimination cannot capture. As the two cohorts differ in center, period and endpoint (in-hospital versus resuscitation-room and 30-day mortality), their agreement on the pH gradient and on discrimination represents external corroboration.

In the resuscitation room, derangements in lactate, pH, and bicarbonate are tightly linked and may collectively reflect acute tissue hypoxia and shock. Elevated lactate levels often indicate the onset of anaerobic metabolism. Prior research has shown that the magnitude of lactate elevation correlates directly with shock severity and mortality across various acute conditions [13, 14]. The results of this study are consistent with these findings, although the adjusted association for lactate was borderline and lost significance when dichotomized, whereas the continuous lactate association remained robust. Lactate should therefore be interpreted with corresponding caution and not presented with the same statistical robustness as OHCA, pH, bicarbonate or hemoglobin. Elevated lactate is commonly interpreted as a marker of tissue hypoxia, though impaired clearance or metabolic dysregulation may also contribute [15].

While other etiologies such as respiratory failure, ketoacidosis, or intoxications may contribute to a low pH in the emergency setting, acidosis, particularly when driven by hyperlactatemia, is generally linked to worse outcomes [16].

A pH < 7.35 was independently associated with mortality (OR 2.26), and low bicarbonate (< 22 mmol/L) showed a similar association (OR 2.79). Progressive consumption of bicarbonate is a physiological consequence of buffering metabolic acids. The absence of problematic collinearity (all VIF ≤ 1.4) indicates that pH and bicarbonate contributed independently to the model rather than compromising its stability, consistent with their partially distinct aspects of acid-base derangement. Both parameters show close arterial-venous agreement in emergency department patients [5–10]. In profound shock, however, venous values may be less reliable, even for pH [6]. Agreement for pCO_2_ is generally less consistent [6]. While data on the predictive role of bicarbonate as an individual parameter in emergency settings remain limited, the findings of this study align with those of Wigger et al., who identified low bicarbonate as an independent predictor of short-term mortality in ICU patients with cardiogenic shock [17]. Although the present study investigated a more heterogeneous cohort at an earlier stage of hospital admission, the results support the hypothesis that low bicarbonate could serve as an additional early indicator of clinical deterioration.

Adequate tissue oxygenation also depends on sufficient oxygen-carrying capacity. Anemia is highly prevalent, affecting approximately one-third of the global population and a substantial proportion of emergency department patients [18, 19]. In the cohort of this study, 28% of patients presented with anemia (hemoglobin < 12 g/dL). While anemia is an established risk factor in general and cardiovascular patients [20–26], data specifically addressing critically ill non-trauma patients in the resuscitation room are limited. The association observed in this study between initial anemia and increased in-hospital mortality (OR 2.07) appears physiologically plausible, as impaired oxygen delivery exacerbates existing shock states. However, anemia may also be a marker of chronic disease burden rather than a direct contributor, and this association should be interpreted accordingly. Similarly, a low bicarbonate may reflect several distinct underlying processes. Given the retrospective design and limited adjustment for illness severity and comorbidity, association rather than mechanism is emphasized.

Glucose displayed a U-shaped rather than monotonic association with mortality. Mortality was highest at the extremes, reaching 40% below 70 mg/dL and 46% above 272 mg/dL, and lowest in the near-normoglycemic range (Fig. 5; Supplementary Table S6), with the spline analysis confirming the non-linear relationship (non-linearity *p* = 0.004). This bidirectional pattern closely reproduces the findings of Bernhard et al. [11], who studied a comparably sized cohort of nontraumatic critically ill patients admitted to a German emergency department resuscitation room with in-hospital mortality as the endpoint and derived the same glucose breakpoints of ≤ 100 and ≥ 272 mg/dL that were adopted a priori in the present study. Relative to their near-normoglycemic reference stratum (101–136 mg/dL, 25.2% mortality), risk in their cohort rose most steeply with hypoglycemia (≤ 100 mg/dL, OR 6.30) and was also elevated with hyperglycemia ≥ 272 mg/dL (272–361 mg/dL, OR 2.53; ≥ 362 mg/dL, OR 2.96). The present data reproduce both arms of this relationship, with comparably high mortality at both extremes. That an independent restricted cubic spline analysis in the present cohort recovered the same U-shape provides external confirmation of these proposed thresholds in a separate patient population. Both a low and a markedly elevated admission glucose most likely reflect how severely ill a patient is rather than directly causing death [27, 28].

The in-hospital mortality rate in this study was 20%, lower than the 30-day mortality of 34% reported by Bernhard et al. in the OBSERvE study [1]. A direct comparison is limited by methodological differences in cohort selection. In the OBSERvE study, 30% of patients with out-of-hospital cardiac arrest were declared dead while still in the resuscitation room. In the present study, patients who died in the resuscitation room or were immediately transitioned to best supportive care were systematically excluded, which likely explains the lower overall mortality rate. Ultimately, the mortality rate in this cohort aligns closely with reported 30-day mortality rates for severe conditions such as sepsis (20–30%) or severe pneumonia (15–34%) [29, 30].

### Limitations

The retrospective, single-center design limits generalizability of the results and precludes any causal inference between initial blood gas derangements and mortality. External validation in independent cohorts is therefore warranted.

A substantial limitation is the high exclusion rate (31.4%) of initially screened patients, primarily due to missing data or insufficient documentation following early inter-hospital transfers. As this exclusion is unlikely to be completely at random, it introduces selection bias. Transferred patients may possess systematically different risk profiles compared to those who remained for the duration of their treatment. Furthermore, excluding patients who deceased directly in the resuscitation room or were immediately transitioned to best supportive care creates a form of survivorship bias. This approach inevitably underestimates the true overall mortality burden of this critically ill demographic. While this exclusion was methodologically intended to focus exclusively on patients requiring ongoing risk stratification for intensive care, it artificially truncates the most severe spectrum of the cohort.

Another substantial limitation of this study is the initial blood gas sample type (arterial, venous, or capillary) could not be reliably reconstructed at the patient level, so a sample-source-stratified analysis was not feasible. This represents a considerable limitation to interpretation of some parameters, because venous-arterial agreement is parameter-dependent and may be affected by critical illness and perfusion abnormalities, even though prior evidence supports acceptable concordance for pH and bicarbonate [5–10].

The categorization of continuous parameters, although implemented for clinical applicability, discards information and may obscure non-linear associations. To address this, all parameters were additionally analyzed continuously and with restricted cubic splines, and the chosen cut-offs are not presented as validated prognostic thresholds. Reassuringly, these cut-off-independent continuous and spline analyses reproduced the principal associations, indicating that the findings do not hinge on the specific thresholds chosen. Only the initial admission blood gas was analyzed. Serial measurements capturing the trajectory of these parameters were unavailable and may carry additional prognostic information. Some strata, notably the glucose extremes, contained few patients, so the corresponding estimates are imprecise, as reflected in their wide confidence intervals. Despite multivariable adjustment, residual confounding remains possible.

Finally, the studied non-trauma cohort is inherently heterogeneous owing to the absence of standardized pre-hospital admission criteria, which introduces additional variability and further complicates the isolation of specific prognostic effects.

## Conclusion

In this retrospective cohort, anemia, acidosis and hypobicarbonatemia on admission were independently associated with an elevated risk of in-hospital mortality in critically ill non-trauma patients. The association for hyperlactatemia was borderline. In the stratified analysis, mortality increased stepwise with decreasing pH. Glucose was associated with higher mortality at both low and high values, with the lowest risk in the near-normal range. These readily available point-of-care parameters may serve as early markers to help identify high-risk patients. Future prospective multicenter studies are needed to confirm these associations and evaluate their utility in guiding early targeted interventions. 

## Supplementary Information

Below is the link to the electronic supplementary material.

Supplementary Material 1

## Data Availability

The data that support the findings of this study are not openly available dueto reasons of sensitivity and are available from the corresponding authorupon reasonable request. Data are located in controlled access data storage atUniversity Hospital Regensburg.

## Declarations

### Use of artificial intelligence

During the preparation of this work, the authors used an AI assistant to improve readability, refine scientific language, and format tables and figures. After using this tool, the authors reviewed and edited the content as needed and take full responsibility for the final content of the publication. The AI tool was not used to generate data, perform primary statistical analysis, or draw scientific conclusions.
