# Supplementary material for: Prognostic value of early point-of-care blood gas analysis in critically ill non-trauma patients
Source: BMC Emerg Med. 2026 Oct 21;26:283. doi: 10.1186/s12873-026-01822-2 (PMC13689306; doi:10.1186/s12873-026-01822-2)
Supplement: Supplementary file 1 [file 12873_2026_1822_MOESM1_ESM.docx]

**Supplementary Material**

**Supplementary Table S1:** Univariate association of initial blood gas and clinical parameters with in-hospital mortality

| Parameter | Total | Deceased | | p-value |
| --- | --- | --- | --- | --- |
|  | n | n | (% [95% CI]) |  |
| pH | 542 | 110 | (20.3% [17.0-23.9]) | < 0.001 |
| 7.35-7.45 | 179 | 13 | (7.3% [3.9-12.1]) | *Ref* |
| < 7.35 | 332 | 95 | (28.6% [23.8-33.8]) | *< 0.001* |
| > 7.45 | 31 | 2 | (6.5% [0.8-21.4]) | *1.000* |
| Base excess | 541 | 110 | (20.3% [17.0-24.0]) | < 0.001 |
| -2-2 mmol/L | 167 | 9 | (5.4% [2.5-10.0]) | *Ref* |
| < -2 mmol/L | 290 | 95 | (32.8% [27.4-38.5]) | *< 0.001* |
| > 2 mmol/L | 84 | 6 | (7.1% [2.7-14.9]) | *0.582* |
| Bicarbonate | 537 | 108 | (20.1% [16.8-23.8]) | < 0.001 |
| 22-28 mmol/L | 248 | 17 | (6.9% [4.0-10.7]) | *Ref* |
| < 22 mmol/L | 266 | 90 | (33.8% [28.2-39.9]) | *< 0.001* |
| > 28 mmol/L | 23 | 1 | (4.3% [0.1-21.9]) | *1.000* |
| Lactate | 541 | 109 | (20.1% [16.8-23.8]) | < 0.001 |
| ≤ 2.2 mmol/L | 244 | 18 | (7.4% [4.4-11.4]) | *Ref* |
| > 2.2 mmol/L | 297 | 91 | (30.6% [25.4-36.2]) | *< 0.001* |
| Glucose | 542 | 110 | (20.3% [17.0-23.9]) | < 0.001 |
| 100-272 mg/dL | 389 | 65 | (16.7% [13.1-20.8]) | *Ref* |
| < 100 mg/dL | 73 | 8 | (11.0% [4.9-20.5]) | *0.293* |
| > 272 mg/dL | 80 | 37 | (46.2% [35.0-57.8]) | *< 0.001* |
| Hemoglobin | 542 | 110 | (20.3% [17.0-23.9]) | 0.002 |
| 12-18 g/dL | 388 | 64 | (16.5% [12.9-20.6]) | *Ref* |
| < 12 g/dL | 149 | 45 | (30.2% [23.0-38.3]) | *< 0.001* |
| > 18 g/dL | 5 | 1 | (20.0% [0.5-71.6]) | *1.000* |
| Sodium | 542 | 110 | (20.3% [17.0-23.9]) | 0.474 |
| 135-145 mmol/L | 427 | 82 | (19.2% [15.6-23.3]) | *Ref* |
| < 135 mmol/L | 83 | 20 | (24.1% [15.4-34.7]) | *0.298* |
| > 145 mmol/L | 32 | 8 | (25.0% [11.5-43.4]) | *0.487* |
| Potassium | 542 | 110 | (20.3% [17.0-23.9]) | 0.185 |
| 3.5-5.5 mmol/L | 427 | 84 | (19.7% [16.0-23.8]) | *Ref* |
| < 3.5 mmol/L | 85 | 16 | (18.8% [11.2-28.8]) | *1.000* |
| > 5.5 mmol/L | 30 | 10 | (33.3% [17.3-52.8]) | *0.099* |
| Calcium | 541 | 110 | (20.3% [17.0-24.0]) | 0.004 |
| 1.15-1.35 mmol/L | 334 | 54 | (16.2% [12.4-20.6]) | *Ref* |
| < 1.15 mmol/L | 195 | 51 | (26.2% [20.1-32.9]) | *0.007* |
| > 1.35 mmol/L | 12 | 5 | (41.7% [15.2-72.3]) | *0.037* |
| Chloride | 541 | 110 | (20.3% [17.0-24.0]) | 0.473 |
| 96-110 mmol/L | 447 | 95 | (21.3% [17.5-25.3]) | *Ref* |
| < 96 mmol/L | 36 | 5 | (13.9% [4.7-29.5]) | *0.393* |
| > 110 mmol/L | 58 | 10 | (17.2% [8.6-29.4]) | *0.606* |
| OHCA | 542 | 110 | (20.3% [17.0-23.9]) | < 0.001 |
| No | 376 | 39 | (10.4% [7.5-13.9]) |  |
| Yes | 166 | 71 | (42.8% [35.1-50.7]) |  |
| Sex | 542 | 110 | (20.3% [17.0-23.9]) | 0.066 |
| Female | 170 | 43 | (25.3% [19.0-32.5]) |  |
| Male | 372 | 67 | (18.0% [14.2-22.3]) |  |
| Age, years, median [IQR] | 542 |  | 67 [53-75] | 0.025 |

Univariate associations of the initial point-of-care blood gas and clinical parameters with in-hospital mortality. Each blood gas parameter is presented as the reference range versus abnormal strata; deceased cases are given as n (%, exact binomial [Clopper-Pearson] 95% CI). P-value at the variables’ top row is the overall Pearson χ² test across strata; an italicized p-value at an abnormal stratum denotes that stratum versus the reference range (Fisher’s exact test). OHCA and Sex were compared by χ² test and Age by the Mann-Whitney U test. A two-sided p < 0.05 was considered significant. CI: confidence interval; IQR: interquartile range; OHCA: out-of-hospital cardiac arrest; Ref: Reference.

**Supplementary Table S2:** Discrimination and calibration of the primary multivariable model

| **Metric** | **Value** |
| --- | --- |
| C-statistic (apparent) | 0.838 (95% CI 0.795-0.880) |
| C-statistic (bootstrap optimism-corrected) | 0.810 |
| Nagelkerke R^2^ | 0.36 |
| Hosmer-Lemeshow test | p = 0.94 |
| Bootstrap optimism (C-statistic) | 0.028 |

Discrimination and calibration of the primary multivariable logistic-regression model. The C-statistic (equivalent to the area under the ROC curve) is reported as the apparent value and after bootstrap optimism correction (200 replications), together with the resulting optimism. Overall fit is summarized by the Nagelkerke R^2^ and calibration by the Hosmer-Lemeshow test (a non-significant p indicates adequate goodness-of-fit). The corresponding ROC and calibration curves are shown in Figure 3 of the manuscript. CI: confidence interval; C-statistic: concordance statistic; R^2^: Nagelkerke pseudo-coefficient of determination; ROC: receiver operating characteristic.

**Supplementary Table S3:** Multicollinearity diagnostics for the multivariable model

| **Variable** | **GVIF** | **df** | **GVIF^(1/2df)** | **VIF-equivalent** |
| --- | --- | --- | --- | --- |
| Age | 1.15 | 1 | 1.07 | 1.15 |
| Sex | 1.04 | 1 | 1.02 | 1.04 |
| OHCA | 1.33 | 1 | 1.15 | 1.32 |
| Calcium | 1.19 | 2 | 1.04 | 1.09 |
| Bicarbonate | 1.50 | 2 | 1.11 | 1.23 |
| Lactate | 1.37 | 1 | 1.17 | 1.37 |
| Glucose | 1.38 | 2 | 1.08 | 1.18 |
| pH | 1.34 | 2 | 1.08 | 1.16 |
| Hemoglobin | 1.19 | 2 | 1.04 | 1.09 |

Multicollinearity diagnostics for the covariates of the primary multivariable model. Collinearity was quantified by the variance inflation factor (VIF); for terms with more than one degree of freedom (multi-category variables) the generalized VIF was rescaled to a VIF-equivalent, [GVIF^(1/2df)]², to allow comparison across terms. All values were well below the conventional threshold of 5, indicating no relevant collinearity among the model covariates. df: degrees of freedom; GVIF: generalized variance inflation factor; OHCA: out-of-hospital cardiac arrest; VIF: variance inflation factor.

**Supplementary Table S4:** Pearson correlations among the continuous acid-base variables

|  | **pH** | **Bicarbonate** | **Base excess** | **Lactate** |
| --- | --- | --- | --- | --- |
| **pH** | 1.00 | 0.75 | 0.73 | -0.62 |
| **Bicarbonate** | 0.75 | 1.00 | 0.98 | -0.67 |
| **Base excess** | 0.73 | 0.98 | 1.00 | -0.68 |
| **Lactate** | -0.62 | -0.67 | -0.68 | 1.00 |

Pearson correlation coefficients (r) among the continuous acid-base variables. Bicarbonate and base excess were near-collinear (r = 0.98); base excess was therefore not entered into the multivariable model. The remaining pairwise correlations were moderate.

**Supplementary Table S5:** Blood gas parameters modelled continuously in relation to in-hospital mortality

| **Parameter** | **OR (95% CI)** | **p (overall)** | **p (non-linearity)** |
| --- | --- | --- | --- |
| pH (per -0.1) | 1.89 (1.62-2.22) | < 0.001 | 0.040 |
| Bicarbonate (per -1 mmol/L) | 1.23 (1.18-1.30) | < 0.001 | 0.24 |
| Base excess (per -1 mmol/L) | 1.15 (1.11-1.19) | < 0.001 | 0.038 |
| Lactate (per +1 mmol/L) | 1.33 (1.26-1.41) | < 0.001 | 0.034 |
| Glucose (per +50 mg/dL) | 1.46 (1.30-1.64) | < 0.001 | 0.004 |
| Hemoglobin (per -1 g/dL) | 1.19 (1.10-1.29) | < 0.001 | 0.45 |
| Sodium (per +1 mmol/L) | 0.99 (0.95-1.02) | 0.31 | 0.22 |
| Potassium (per +0.5 mmol/L) | 1.12 (1.00-1.25) | 0.051 | 0.15 |
| Calcium (per +0.1 mmol/L) | 0.76 (0.61-0.94) | < 0.001 | 0.001 |
| Chloride (per +1 mmol/L) | 0.98 (0.94-1.01) | 0.002 | 0.004 |

Blood gas parameters modelled continuously in relation to in-hospital mortality. Odds ratios per unit change from univariable logistic regression. P (overall) and p (non-linearity) are from a restricted cubic spline (4-knot) likelihood-ratio test. Where non-linearity is significant, the per-unit odds ratio should be interpreted as a linear approximation of a non-monotone association. Base excess was collinear with bicarbonate (Supplementary Table S4) and was therefore not entered into the multivariable model. Sodium, potassium and chloride are reported for completeness and are not displayed in Figure 4: sodium and potassium were not significantly associated with mortality, and the chloride association, although statistically significant, was of negligible magnitude (OR 0.98 per mmol/L). CI: confidence interval; OR: odds ratio.

**Supplementary Table S6:** In-hospital mortality across graded pH and glucose strata

| **pH stratum** | **n** | | **Deaths** | **Mortality (95% CI)** |
| --- | --- | --- | --- | --- |
| < 7.0 | 29 | | 19 | 65.5% (45.7-82.1) |
| 7.0-7.2 | 84 | | 36 | 42.9% (32.1-54.1) |
| 7.2-7.35 | 219 | | 40 | 18.3% (13.4-24.0) |
| 7.35-7.45 | 179 | | 13 | 7.3% (3.9-12.1) |
| > 7.45 | 31 | | 2 | 6.5% (0.8-21.4) |
|  | | | | |
| **Glucose stratum (mg/dL)** | | **n** | **Deaths** | **Mortality (95% CI)** |
| < 70 | 10 | | 4 | 40.0% (12.2-73.8) |
| 70-100 | 63 | | 4 | 6.3% (1.8-15.5) |
| 100-140 | 172 | | 16 | 9.3% (5.4-14.7) |
| 140-200 | 120 | | 19 | 15.8% (9.8-23.6) |
| 200-272 | 97 | | 30 | 30.9% (21.9-41.1) |
| > 272 | 80 | | 37 | 46.2% (35.0-57.8) |

**Upper panel (pH strata):** Observed in-hospital mortality across graded pH strata, showing the number at risk (n), the number of deaths, and the mortality proportion (exact binomial 95% CI). Mortality increased significantly as pH fell (test for trend, p < 0.001).

**Lower panel (Glucose strata):** Observed in-hospital mortality across graded strata of admission glucose, showing the number at risk (n), the number of deaths, and the mortality proportion (exact binomial 95% CI). Mortality differed significantly across strata (p < 0.001) and followed a U-shaped pattern with a nadir at 70-100 mg/dL (test for a quadratic component, p = 0.006).

CI: confidence interval.

**Supplementary Table S7:** Sensitivity analysis: multivariable model re-including resuscitation-room deaths (expanded cohort)

| Parameter |  | Primary OR  (95% CI) | Primary p-value | Expanded OR (95% CI) | Expanded p-value |
| --- | --- | --- | --- | --- | --- |
| Age | per year | 1.03 (1.01-1.04) | 0.003 | 1.03 (1.01-1.05) | < 0.001 |
| Sex | Male | Reference |  | Reference |  |
|  | Female | 1.51 (0.89-2.57) | 0.124 | 1.43 (0.85-2.39) | 0.174 |
| OHCA | No | Reference |  | Reference |  |
|  | Yes | 3.38 (1.92-6.04) | < 0.001 | 4.05 (2.34-7.12) | < 0.001 |
| pH | 7.35-7.45 | Reference |  | Reference |  |
|  | < 7.35 | 2.26 (1.09-4.95) | 0.033 | 1.87 (0.94-3.85) | 0.081 |
|  | > 7.45 | 1.10 (0.15-4.95) | 0.914 | 0.83 (0.12-3.61) | 0.820 |
| Bicarbonate | 22-28 mmol/L | Reference |  | Reference |  |
|  | < 22 mmol/L | 2.79 (1.42-5.69) | 0.004 | 2.47 (1.29-4.84) | 0.007 |
|  | > 28 mmol/L | 0.60 (0.03-3.62) | 0.645 | 0.52 (0.03-3.04) | 0.548 |
| Lactate | ≤ 2.2 mmol/L | Reference |  | Reference |  |
|  | > 2.2 mmol/L | 1.85 (0.93-3.77) | 0.081 | 1.88 (0.97-3.71) | 0.063 |
| Glucose | 100-272 mg/dL | Reference |  | Reference |  |
|  | < 100 mg/dL | 1.15 (0.41-2.94) | 0.784 | 1.60 (0.63-3.79) | 0.302 |
|  | > 272 mg/dL | 1.72 (0.93-3.19) | 0.084 | 1.72 (0.94-3.13) | 0.075 |
| Hemoglobin | 12-18 g/dL | Reference |  | Reference |  |
|  | < 12 g/dL | 2.07 (1.20-3.58) | 0.009 | 1.97 (1.17-3.33) | 0.011 |
|  | > 18 g/dL | 0.80 (0.04-7.47) | 0.854 | 1.66 (0.19-11.52) | 0.611 |
| Calcium | 1.15-1.35 mmol/L | Reference |  | Reference |  |
|  | < 1.15 mmol/L | 0.91 (0.53-1.54) | 0.725 | 0.82 (0.49-1.36) | 0.452 |
|  | > 1.35 mmol/L | 3.83 (0.82-17.14) | 0.080 | 2.85 (0.63-12.23) | 0.160 |

Sensitivity analysis comparing the primary and expanded multivariable models. Both columns are complete-case multivariable logistic-regression models with profile-likelihood confidence intervals, corresponding to main-text Table 3. Within each parameter the normal reference range is shown as the reference category and the abnormal strata as adjusted odds ratios. The expanded model re-includes the 13 resuscitation-room deaths that had a complete initial blood gas panel and a consistently recorded outcome, increasing the cohort from 542 (110 deaths) to 555 (123 deaths, 22.2%) and the fitted sample from n = 535 to n = 548. Conclusions were unchanged; the association with out-of-hospital cardiac arrest was stronger, whereas the association with low pH was attenuated to borderline significance (OR 1.87, p = 0.081). CI: confidence interval; OHCA: out-of-hospital cardiac arrest; OR: odds ratio.

**Supplementary Table S8:** Sensitivity analysis of the continuous parameters in the expanded cohort

| Parameter | OR (95% CI) | p (overall) | p (non-linearity) |
| --- | --- | --- | --- |
| pH (per -0.1) | 1.94 (1.68-2.26) | < 0.001 | 0.19 |
| Bicarbonate (per -1 mmol/L) | 1.24 (1.18-1.30) | < 0.001 | 0.66 |
| Base excess (per -1 mmol/L) | 1.15 (1.11-1.19) | < 0.001 | 0.21 |
| Lactate (per +1 mmol/L) | 1.33 (1.26-1.41) | < 0.001 | 0.075 |
| Glucose (per +50 mg/dL) | 1.45 (1.30-1.63) | < 0.001 | 0.002 |
| Hemoglobin (per -1 g/dL) | 1.18 (1.10-1.27) | < 0.001 | 0.19 |
| Sodium (per +1 mmol/L) | 0.99 (0.95-1.02) | 0.39 | 0.29 |
| Potassium (per +0.5 mmol/L) | 1.16 (1.04-1.28) | 0.006 | 0.087 |
| Calcium (per +0.1 mmol/L) | 0.81 (0.66-1.00) | 0.001 | 0.002 |
| Chloride (per +1 mmol/L) | 0.97 (0.94-1.00) | 0.002 | 0.007 |

Sensitivity analysis of the continuous parameters in the expanded cohort (n = 555, 123 deaths), re-including the 13 resuscitation-room deaths with a complete initial blood gas panel and a consistently recorded outcome; otherwise analyzed as in Supplementary Table S5. Odds ratios are per unit change from univariable logistic regression with each parameter modelled continuously; p (overall) and p (non-linearity) are from restricted cubic spline (four-knot) likelihood-ratio tests. Estimates were essentially identical to the primary cohort (Supplementary Table S5), and the U-shaped glucose-mortality association persisted (non-linearity p = 0.002). CI: confidence interval; OR: odds ratio.
